# Ventilator-Associated Pneumonia in COVID-19 Patients: A Retrospective Cohort Study

**DOI:** 10.3390/antibiotics10080988

**Published:** 2021-08-16

**Authors:** Maxence Rouyer, Alessio Strazzulla, Tracie Youbong, Paul Tarteret, Aurélia Pitsch, Astrid de Pontfarcy, Bruno Cassard, Nicolas Vignier, Franck Pourcine, Sébastien Jochmans, Mehran Monchi, Sylvain Diamantis

**Affiliations:** 1 Infectious Diseases Unit, Groupe Hospitalier Sud Ile de France, 77000 Melun, France; maxence.rouyer@ghsif.fr (M.R.); youbsnet@gmail.com (T.Y.); paultarteret@gmail.com (P.T.); astrid.depontfarcy@ghsif.fr (A.d.P.); nicolas.vignier@ghsif.fr (N.V.); sylvain.diamantis@ghsif.fr (S.D.); 2 Internal Medicine Unit, Groupe Hospitalier Sud Ile de France, 77000 Melun, France; 3 Medical Biology Laboratory, Groupe Hospitalier Sud Ile de France, 77000 Melun, France; Aurelia.Pitsch@ghsif.fr; 4 Pharmacy Unit, Groupe Hospitalier Sud Ile de France, 77000 Melun, France; cassard.bruno@ghsif.fr; 5 Intensive Care Unit, Groupe Hospitalier Sud Ile de France, 77000 Melun, France; frank.pourcine@ghsif.fr (F.P.); sebastien.jochmans@ghsif.fr (S.J.); mehran.monchi@ghsif.fr (M.M.)

**Keywords:** blood culture, coronavirus infectious disease 2019, polymicrobial culture, severe acute respiratory syndrome-coronavirus 2, ventilator-associated pneumonia

## Abstract

Introduction: Aim of this study is to analyse the characteristics of ventilator-associated pneumonia (VAP) inpatients infected by severe acute respiratory syndrome-coronavirus 2 (SARS-CoV-2). Materials and Methods: A retrospective study was conducted, including coronavirus infectious disease 2019 (COVID-19) patients who developed VAP from March to May 2020 (VAP COVID-19). They were compared to non-COVID-19 patients who developed VAP from January 2011 to December 2019 (VAP NO COVID-19) and COVID-19 patients who did not develop VAP (NO VAP COVID-19). Results: Overall, 42 patients were included in the VAP COVID-19group, 37 in the NO VAP COVID-19 group, and 188 in the VAP NO COVID-19 group. VAP COVID-19 had significantly higher rates of shock (71% vs. 48%, *p* = 0.009), death in ICU (52% vs. 30%, *p* = 0.011), VAP recurrence (28% vs. 4%, *p* < 0.0001), positive blood culture (26% vs. 13%, *p* = 0.038), and polymicrobial culture (28% vs. 13%, *p* = 0.011) than VAP NO COVID-19. At the multivariate analysis, death in patients with VAP was associated with shock (*p* = 0.032) and SARS-CoV-2 (*p* = 0.008) infection. Conclusions: VAP in COVID-19 patients is associated with shock, bloodstream, and polymicrobial infections.

## 1. Introduction

The coronavirus infectious disease 2019 (COVID-19) pandemic caused 172,630,637 confirmed casesworldwide, including 3,718,683 deaths by 6 June 2021 [1]. Overall, almost 25% of COVID-19 patients required critical care, and, therefore, they were hospitalised in intensive care units (ICUs) [2]. Because the recurrence to mechanical ventilation is frequent, these patients are at risk of developing ventilator-associated pneumonia (VAP) [3].

VAP is defined as an infection of pulmonary parenchyma that develops in patients receiving mechanical ventilation for at least 48 h [4]. VAP is a life-threatening disease associated with high mortality rates (43%) [5]. It is sustained by different microorganisms, especially Staphylococcus aureus, Enterobacteriaceae, and non-fermenting Gram-negative bacteria (*Pseudomonas aeruginosa*, *Acinetobacter baumannii*, and *Stenotrophomonas maltophilia*) [4]. The main risk factors for VAP are: advanced age, male gender, increased duration of mechanical ventilation, prolonged length of hospital stay, multiple trauma, sepsis, central nervous diseases, burns, previous antibiotic treatment, smoking, and invasive medical procedures of the respiratory tract [6,7].

VAP among COVID-19 patients has already been investigated. According to different studies, its incidencefluctuated from 36% to 85%, and mortality rates in ICUs varied from 29% to 43% [3,8,9,10,11]. Moreover, VAP in COVID-19 is associated with increased 28-day mortality [12]. The main factors associated with mortality during VAP are septic shock and severe acute respiratory syndrome (ARDS). However, features and specific risk factors of VAP in COVID-19 patients have not yet been established [13].

The aim of this study is to explore the differences between VAP in COVID-19 and non-COVID-19 patients in terms of clinical, microbiological, and biochemical characteristics. The main hypothesis to verify is that VAP in COVID-19 is a new “pathology” with some peculiarity that needs different healthcare than VAP in non-COVID-19 patients.

## 2. Materials and Methods

A monocentric retrospective cohort study was conducted in the ICU of a 350 acute-care bed hospital in the Ile de France region in France. All COVID-19 patients who developed VAP inthe ICU from 1 March 2020 to 1 May 2020 were included (VAP COVID-19 group). This population was compared with two other populations: (i) all non-COVID-19 patients who developed VAP during hospitalization in the ICU from 1 January 2011 to 31 December 2019 (VAP NO COVID-19 group); (ii) all COVID-19 patients receiving mechanical ventilation who did not develop VAP during hospitalization in ICU from 1 March 2020 to 1 May 2020 (NO VAP COVID-19 group). The choice of comparing two different timeframes was motivated by the following: (i) during the COVID-19 pandemic, the ICU of our hospital was exclusively reserved forCOVID-19 patients; (ii) VAP in non-COVID-19 patients is less frequent than COVID-19 patients, and our ICU’s capacity is limited. For these reasons, we were forced to select non-COVID-19 patients during a longer timeframe.

The study was conducted in accordance with the Declaration of Helsinki and national and institutional standards. According to French law, approval by the local ethics committee was not necessary because of the non-interventional design of the study. Similarly, the non-interventional nature of the study required only the absence of patients’ opposition. For this reason, a written consent form was not proposed [14,15].

For the definition of COVID cases, only severe acute respiratory syndrome-coronavirus 2 (SARS-CoV-2)-confirmed COVID-19 cases were included in the study, in accordance with international recommendations [16]. Analysis of SARS-CoV-2 genotypes was not performed.

VAP is defined as an infection of pulmonary parenchyma developed after at least 48 h of mechanical ventilation [4]. To achieve a VAP diagnosis, all patients suspected of having VAP will receive alveolar bronchoscopy, and bacterial cultures are obtained from bronchoalveolar lavage (BAL). VAP is suspected when patients developtwo of the following symptoms and signs after at least 48 h of mechanical ventilation: (i) new onset of fever; (ii) purulent endotracheal aspirate; (iii) leukocytosis or leucopenia; (iv) increased minute ventilation; (v) arterial oxygenation decline; (vi) need for increased vasopressor infusion to maintain blood pressure; (vii) new or progressive persistent infiltrate on chest radiograph or computed tomography. VAP is confirmed when bacterial cultures from BAL are positive and have significant quantitative growth (at least 10^4^ colony-forming units/mL) [17].

Recurrence is defined when clinical signs of VAP appear at least 72 h after the end of antibiotic treatment of a previous VAP and after the clinical resolution of VAP. The same clinical and microbiological criteria already used for the definition of VAP are applied. VAP recurrence includes: (i) relapse by the same causative bacterial strains of previous VAP; (ii) superinfection by causative bacterial strains different than previous VAP [9]. Resolution is defined as the normalization of these parameters: body temperature ≤ 37.5 degrees Celsius, leukocytes count ≤ 10 G/L, PaO2/FiO2 ratio ≥ 25 kPa, absence of bacterial growth in cultures from the lower respiratory tract [18].

Patient characteristics, laboratory data, and clinical outcomes were collected through the software used in routinely daily activity: Sillage v17.2.4.5 and CGM Lab channel 1.20.33686. Patient characteristics included: age, gender, body mass index (BMI), co-morbidities (diabetes, heart failure, liver cirrhosis, neoplasia, severe kidney disease, stroke, chronic obstructive pulmonary disease, or COPD), immunosuppressive treatments, antibiotic treatment before the onset of VAP, multidrug-resistant (MDR) bacterial colonisation, simplified acute physiology score II (SAPS-II), ARDS, time of VAP onset from orotracheal intubation (OTI), shock, cultures from lower respiratory tract samples, and blood cultures, treatments (antibiotics, corticosteroids, extracorporeal blood purification). Severe kidney disease was defined for estimated glomerular filtration rate (eGFR) < 30 mL/min [19]. An onset of VAP ≤ 96 h from the start of mechanical ventilation was considered for the definition of early VAP [20]. Shock was defined by the need for vasopressors to maintain a mean arterial pressure ≥ 65 mmHg at VAP onset [21]. Patients receiving invasive or non-invasive ventilation with PaO_2_/FiO_2_ lower than 300 mm Hg were considered to have ARDS, according to Berlin’s criteria [22]. MDR bacterial acquisition was defined according to the results of nasopharyngeal and rectal swabs (obtained at admission and discharge).

The main outcome was death in ICU. Secondary outcomes were death at the end of antibiotic treatment, in-hospital death, duration of OTI, length of hospital stay, length of antibiotic treatment, MDR bacterial acquisition, and clinical improvement at days 3 and 7 of antibiotic treatment. For the latter outcome, the judgement was performed by a multidisciplinary board constituted by an intensive care specialist and an infectious diseases specialist. Clinical improvement was defined as the combined resolution of signs and symptoms of infection, improvement of oxygenation parameters, no increase of the sequential organ failure assessmentscore, and the resolution or stability of radiological images [23].

Statistical analysis was performed using Epi Info^®^ 7.1 (CDC, DeKalb, GA, USA) and SPSS 20 (IBM, Armonk, NY, USA). Statistical significance was set at *p* < 0.050.

For univariate analysis, Fisher’s exact test (qualitative variables) and Student’s *t*-test (quantitative variables) were used. Quantitative variables were presented in the text as median values. For outcomes and clinical characteristics, the following comparisons were made: (i) VAP COVID-19 vs. VAP NO COVID-19; (ii) VAP COVID-19 vs. NO VAP COVID-19 (descriptive analysis). For ARDS, a statistical test was not applied because the presence of ARDS was a mandatory criterion for hospitalisation in the ICU of COVID-19 patients during the SARS-CoV-2 pandemic. Therefore, a statistical test would have been biased.

Multiple logistic regression analysis was performed to explore the characteristics associated with death in ICU among patients who developed VAP with and without SARS-CoV-2 infection. Parameters included in the multivariate analysis were chosen according to univariate analysis results (*p* ≤ 0.05). Included were: BMI, cirrhosis, eGFR < 30 mL/min, heart failure, polymicrobic culture, positive blood culture, shock, and SARS-CoV-2 infection. Some significant variables at univariate analysis (*p* ≤ 0.05) were not included: (i) immunosuppressive treatments, because the beneficial or harmful role of these treatments in SARS-CoV-2 controversial infections is still unknown, and in some cases, they were administrated as a therapeutic drug for SARS-CoV-2; (ii) previous antibiotic treatment in ICU and MDR colonisation at admission, because they were all linked with the variables ESBL *Enterobacteriacae* and MRSA, which were not significant at univariate analysis; (iii) *Haemophilus influenzae* and *Streptococcus* spp., because the study population was not large enough to explore the impact of each single bacterial species.

## 3. Results

From 1 March 2020 to 1 May 2020, 346 patients were hospitalised inthe ICU. Among them, 100/346 (29%) were SARS-CoV-2 PCR-positive, 79/100 (79%) received mechanical ventilation,42/79 (53%) experienced VAP, and 12/42 (28%) had a VAP relapse. From 1 January 2011 to 31 December 2019, 188 patients experienced VAP in the ICU, and 8/188 (4%) had VAP relapse.

Table 1 compares characteristics of the two populations of patients experiencing VAP (VAP COVID-19 vs. VAP NO COVID-19). COVID-19 patients experiencing VAP had significantly higher rates of shock (71% vs. 48%, *p* = 0.009), death in ICU (52% vs. 30%, *p* = 0.011), VAP recurrence (28% vs. 4%, *p* < 0.0001), clinical worsening at day 3 (81% vs. 32%, *p* < 0.0001) and 7 (83% vs. 28%, *p* < 0.0001), positive blood culture (26% vs. 13%, *p* = 0.038), and polymicrobial culture (28% vs. 13%, *p* = 0.011) than non-COVID-19 patients.

At the multivariate analysis, death in ICU among patients who developed VAP was associated with shock (*p* = 0.032) and SARS-CoV-2 (*p* = 0.008), as shown in Table 2.

Table 3 presents a descriptive analysis of characteristics of COVID-19 patients receiving mechanical ventilation who experienced VAP or not during hospitalisation in the ICU (VAP COVID-19 vs. NO VAP COVID-19).

## 4. Discussion

This study showed that VAP was frequent in COVID-19 patients receiving mechanical ventilation, andit was associated with previous antibiotic treatment and shock.Its morbidity was principally due to SARS-CoV-2 infection and some other microbiologic characteristics, such as an association with positive blood cultures and polymicrobial cultures rather than patients’ related risk factors, such as immune-depression and co-morbidities.As expected, VAP in COVID-19 patients was associated with prolonged OTI and length of hospital stay.

In our study, VAP occurred in more than 50% of COVID-19 patients receiving mechanical ventilation, and it was associated with death in 50% of cases.These results are aligned with data presented by other authors [8,9,10]. Additionally, no significant difference in mortality rate was detected between COVID-19 patients with and without VAP. This result is encouraging, and it suggests that VAP can be managed in ICUs, and the risk of VAP should not be a limitation to mechanical ventilation in COVID-19 patients. Unfortunately, our study design did not allow us to explore which factors could increase the risk of VAP in COVID-19 patients. Among these factors, it would be interesting to explore whether the use of aerosol generation personal protective equipment (AGPPE) could have influenced rates of VAP during the different waves of the COVID pandemic. Indeed, it is possible that the use of AGPPE could influence not only SARS-CoV-2 transmissions but also the transmission of other microorganisms in a positive way (reducing contact with potentially contaminated surfaces) or negative way (reducing attention to patient hygiene). Additionally, the level of preparation of healthcare personnel in the use of AGPPE, as well as the level of alert felt by healthcare workers, could impact VAP rates in COVID-19 (and non-COVID-19) patients.

The high levels of antibiotic treatment administrated before VAP onset (73% in COVID-19 patients and 88% in non-COVID-19 patients) confirmed that antibiotic administration is a risk factor for VAP onset. Additionally, COVID-19 patients presented a higher rate of MDR bacterial colonisation at admission toICU (31% vs. 16%) but they didnot have a higher risk of MDR bacterial acquisition during hospitalisation in ICU. We can speculate that the reason for MDR carriage was the high antibiotic intake observed in France during the COVID-19 pandemic in the early months of 2020, especially for azithromycin [24]. On the other side, the measures of antimicrobial stewardship actually applied in our ICU service could have limited the risk of in-hospital MDR bacterial acquisition [25,26,27,28]. Therefore, antibiotic treatment should be proposed only in COVID-19 patients with documented bacterial infection to limit the risk of VAP caused by MDR bacteria [29,30,31,32].

The clinical evolution was worse in COVID-19 patients than non-COVID-19 patients affected by VAP. The higher mortality observed could be explained by the SARS-CoV-2 infection and the higher rates of shock (71% vs. 48%), as also demonstrated by other authors [33]. Shock could have been enhanced by ARDS, which was present in 100% of COVID-19 patients and only in 59% of non-COVID-19 patients. However, the study design did not allow us to investigate the role of SARS-CoV-2 and bacterial pulmonary infection in causing shock and ARDS.

A particular aspect of VAP in COVID-19 patients was its association with positive blood cultures. This result implied some consequences. First of all, it could explain the higher rate of shock in COVID-19 patients with VAP. Secondly, it justifies a different strategy of antibiotic treatment of VAP, with molecules having not only good penetration in pulmonary parenchyma but also a high blood distribution. Additionally, an association of two antibiotics could be argued. This is the first study toreport such an association, to our best knowledge, and its results need to be confirmed in further studies.Finally, the association of VAP with positive blood cultures is not a veritable surprise, considering the high frequency of bloodstream infections in COVID-19 patients hospitalised in ICU [34].

Another unusual characteristic of VAP in COVID-19 patients was the high rate of polymicrobial infections (28%). This result was in accordance with data presented by Luyt et al. (30%)but sensibly higher than data presented by Rouzé et al. (9.8%) [8,9]. Polymicrobial pulmonary infections are more difficult to treat than monomicrobial infections [33,35]. The association of polymicrobial infection with VAP in COVID-19 patients could partially explain the higher incidence of VAP recurrence observed among COVID-19 patients rather than non-COVID-19 patients (28% vs. 4%). We think that the risk of polymicrobial infections has to be kept in mind when an empirical antibiotic treatment is prescribed for VAP in COVID-19 patients.

When clinical characteristics of patients were compared, we found that COVID-19 patients who developed VAP had less co-morbidity than non-COVID-19 patients and the same rates of co-morbidities asCOVID-19 patients who did not develop VAP. These results were important, and they depicteda different scenario for VAP during COVID-19. Indeed, VAP developed in patients that were generally healthy before hospitalisation, and they were rapidly weakened by an immunological storm caused by the SARS-CoV-2 infection. The lower rate of co-morbidities could have partially compensatedfor the severity of COVID-19, and it could have reduced the mortality rate of VAP in COVID-19 patients.

Finally, we found that length of stay and duration of OTI were longer in COVID-19 patients with VAP than in COVID-19 patients without VAP.As a consequence, each measure to reduce the risk of VAP should be enforced to facilitate patient turnover in ICUs. However, the study design made it impossible to analyse whether prolonged OTI and length of stay were actually the cause or the consequence of VAP.

This study presents several limitations: (i) a certain amount of missing data is predictable because a retrospective cohort study was conducted; (ii) data about clinical safety were not completelycollected in the medical software. This lack of “analogic” data limited the analysis. In particular, an exhaustive sub-analysis of factors associated with shock was not possible; (iii) this was a monocentric study; thus, its conclusions cannot be directly applied to other centres.

## 5. Conclusions

For the first time, this study made a direct comparison between VAP in COVID-19 patients and non-COVID-19 patients. These two pathologies do not coincide at 100% because of the different backgrounds constituted by the SARS-CoV-2 infection and its consequence in terms of inflammation and pathogenicity. Results of this study will contribute to improving the healthcare of VAP in COVID-19 patients.

VAP in COVID-19 patients is frequent, and it has some particular characteristics. It is often associated with shock, the nature of which needs to be explored in further studies. From the microbiological point of view, its association with bloodstream and polymicrobial infections needs to be considered during the prescription of antibiotic treatment, either empiric or targeted, to limit the risk of treatment failure and VAP recurrence. Finally, immune-depression and co-morbidities did not appear as key factors for the development of VAP among COVID-19 patients.

## Figures and Tables

**Table 1:** Characteristics of VAP occurring in patients with or without SARS-CoV-2 infection.

-	Characteristics	SARS-CoV-2		*p*-Value
		Yes	No	-
		*n* = 42	*n* = 188	-
**Patients’ Characteristics**	**Biological Characteristics**	-	-	-
	Age (years), mean (SD)	60 (9.7)	634 (13.9)	0.061
	Male gender, *n* (%)	28 (67)	138 (73)	0.446
	BMI, mean (SD)	31 (6.5)	28 (6.4)	0.017
	**Co-Morbidities**	-	-	-
	Diabetes, *n* (%)	10 (24)	58 (31)	0.455
	Heart failure, *n* (%)	6 (14)	92 (48)	<0.0001
	Liver cirrhosis, *n* (%)	1 (2)	34 (18)	0.007
	Neoplasia, *n* (%)	2 (5)	29 (15)	0.081
	eGFR <30, *n* (%)	1 (2)	30 (16)	0.013
	**Risk Factors of Severity**	-	-	-
	Immunosuppressive treatments *, *n* (%)	2 (5)	48 (26)	0.002
	Antibiotic treatment during the last 3 months, *n* (%)	12 (29)	64 (35)	0.474
	Previous antibiotic treatment in ICU, *n* (%)	31 (74)	161 (88)	0.028
	MDR bacterial colonisation at admission, *n* (%)	13 (31)	24 (16)	0.043
	**Reason for ICU Admission**	-	-	-
	Cardiac arrest, *n* (%)	0 (0)	2 (1)	-
	Shock, *n* (%)	0 (0)	22 (12)	-
	ARDS, *n* (%)	42 (100)	63 (33)	NA **
	Gastrointestinal bleeding, *n* (%)	0 (0)	5 (3)	-
	Impaired consciousness, *n* (%)	0 (0)	21 (11)	-
	Others, *n* (%)	0 (0)	16 (9)	-
	Unknown, *n* (%)	0 (0)	59 (31)	-
**VAP Characteristics**	**Clinical Characteristics**	-	-	-
	SAPS-II, mean (SD)	44 (15.7)	49 (17.5)	0.085
	ARDS, *n* (%)	42 (100)	79 (59)	NA **
	Shock, n (%)	29 (71)	91 (48)	0.0009
	VAP onset from OTI (days), mean (SD)	8 (6,7)	9 (6.3)	0.521
	Early VAP, *n* (%)	18 (43)	73 (40)	0.9
	Positive blood culture ***, *n* (%)	11 (26)	21 (13)	0.038
	Polymicrobial culture, *n* (%)	12 (28)	24 (13)	0.011
	**Microbiological Isolates**	-	-	-
	Enterobactériacae, *n* (%)	23 (55)	113 (60)	0.551
	Pseudomonas aeruginosa, *n* (%)	8 (19)	44 (23)	0.554
	Other Gram-negative bacteria, *n* (%)	7	20 (10)	0.288
	Gram-positive bacteria, *n* (%)	12 (29)	23 (17)	0.015
	**First-Line Antibiotic Treatment**	-	-	-
	Amoxicillin ± clavulanic acid, *n* (%)	10 (26)	35 (19)	0.228
	Piperacillin ± tazobactam, *n* (%)	18 (46%)	66 (36)	0.274
	Other single molecule, *n* (%)	19 (49)	95 (52)	0.860
	Association of ≥2 molecules, *n* (%)	8 (21)	26 (14)	0.327
	**Targeted Treatment**	-	-	-
	Amoxicillin ± clavulanic acid, *n* (%)	4 (10)	12 (6)	0.499
	Piperacillin ± tazobactam, *n* (%)	4 (10)	32 (17)	0.344
	Other single molecule, *n* (%)	34 (83)	149 (81)	0.828
	Association of ≥2 molecules, *n* (%)	3 (7)	29 (16)	0.218
	**Other Treatments**	-	-	-
	Corticosteroids, *n* (%)	15 (35)	11 (15)	0.051
	Extracorporeal blood purification, *n* (%)	7 (17)	5 (14)	0.765
**Outcomes**	**Clinical Outcomes**	-	-	-
	MDR bacterial acquisition, *n* (%)	11 (27)	20 (23)	0.637
	Death at EoT, *n* (%)	12 (29)	34 (18)	0.137
	Death in ICU, *n* (%)	22 (52)	54 (30)	0.011
	VAP recurrence, *n* (%)	12 (28)	8 (4)	<0.0001
	Clinical improvement at day 3, *n* (%)	7 (19)	116 (68)	<0.0001
	Clinical improvement at day 7, *n* (%)	7 (17)	95 (72)	<0.0001
	**Other Outcomes**	-	-	-
	Length of hospital stay (days), mean (SD)	33 (22.0)	30 (29.2)	0.391
	Length of antibiotic treatment (days), mean (SD)	7 (3.2)	7 (3.6)	0.121

* Except for corticosteroid treatment; ** statistical test was not applicable because ARDS was a mandatory criterion for hospitalisation in ICU for SARS-CoV-2 patients; *** considering only positive blood culture of germs already isolated from lower respiratory tract samples; ARDS = acute respiratory distress syndrome; BMI = body mass index; eGFR = estimated glomerular filtration rate; EoT = end of treatment; ESBL = extended spectrum beta-lactamase;ICU = intensive care unit; MDR = multidrug resistant; MRSA = methicillin resistant *Staphylococcus aureus*; NA = not applicable; OTI = orotracheal intubation; SAPS-II = simplified acute physiology score-2; SARS-CoV-2 = severe acute respiratory syndrome coronavirus 2; SD = standard deviation; VAP = ventilator-associated pneumonia.

**Table 2:** Multivariate analysis of factors associated with death in the intensive care unit among patients affected by ventilator-associated pneumonia with or without SARS-CoV-2.

Parameter	Or (95%CI)	*p*-Value
BMI	0.992 (0.506–1.945)	0.559
Cirrhosis	1.465 (0.600–3.572)	0.244
eGFR < 30 mL/min	0.761 (0.295–1.960)	0.708
Heart failure	1.419 (0.698–2.848)	0.070
Polymicrobic culture	0.902 (0.304–2.122)	0.535
Positive blood culture	2.172 (0.929–5.021)	0.206
SARS-CoV-2	3.309 (1.369–7.996)	0.008
Shock	2.321 (1.196–4.502)	0.032

BMI = body mass index; eGFR = estimated glomerular filtration rate; SARS-CoV-2 = severe acute respiratory syndrome-coronavirus 2.

**Table 3:** Characteristics of patients with SARS-CoV-2 infection receiving mechanical ventilation and experiencing or not experiencing ventilator-associated pneumonia.

-	Characteristics	VAP	
		Yes	No
		*n* = 42	*n* = 37
**Patient’s Characteristics**	**Biological Characteristics**	-	-
	Age, mean (SD)	60 (9.8)	64 (12.5)
	Male Gender, *n* (%)	28 (67)	27 (73)
	BMI, mean (SD)	31 (6.2)	30 (5.5)
	Non-caucasian, *n* (%)	27 (64)	22 (59)
	**Co-Morbidities**	-	-
	Diabetes, *n* (%)	10 (24)	13 (35.1)
	Arterial hypertension, *n* (%)	26 (62)	25 (67.6)
	Stroke (%)	0(0)	4 (10.8)
	Heart failure, *n* (%)	6 (14)	3 (8.1)
	eGFR <30, *n* (%)	1 (2.4)	NA
	Liver cirrhosis, *n* (%)	1 (2)	0 (0)
	COPD, *n* (%)	5 (12)	5 (14)
	Solid neoplasia, *n* (%)	2 (5)	2 (5)
	Haemopathy, *n* (*n*)	0 (0)	2 (5)
**COVID-19 Characteristics**	**Clinical Characteristics**	-	-
	Corticosteroid treatment, *n* (%)	15 (35)	16 (43)
	SAPS-II, mean (SD)	43 (13.3)	47 (16.9)
	**Biochemical Parameters**	-	-
	C-reactive protein (mg/L), mean (SD)	190 (105.7)	179 (105.8)
	Lactate dehydrogenase (U/L), mean (SD)	566 (285.9)	537 (193.0)
	Lymphocytes (G/L), mean (SD)	0.7 (0.3)	0.9 (0.4)
	Neutrophils (G/L), mean (SD)	8.3 (3.6)	7.2 (4.0)
**Outcomes**	**Death**	-	-
	Death in ICU, *n* (%)	22 (52)	16 (43)
	In-hospital death, *n* (%)	21 (50)	16 (43)
	**Other Outcomes**	-	-
	Duration of OTI (days), mean (SD)	27 (21.6)	12 (9.2)
	Length of hospital stay (days), mean (SD)	31 (21.0)	13 (9.8)

BMI = body mass index; COPD = chronic obstructive pulmonary disease; eGFR = estimated glomerular filtration rate; ICU = intensive care unit; NA = not applicable; OTI = orotracheal intubation; SAPS-II = simplified acute physiology score-2; SARS-CoV-2 = severe acute respiratory syndrome coronavirus 2; SD = standard deviation; VAP = ventilator-associated pneumonia.

## Data Availability

Data is contained within the article.

## Abbreviations

AGPPE	aerosol generation personal protective equipment
ARDS	severe acute respiratory syndrome
BAL	bronchoalveolar lavage
BMI	body mass index
COPD	chronic obstructive pulmonary disease
COVID-19	coronavirus infectious disease 2019
eGFR	glomerular filtration rate
ICU	intensive care unit
MDR	multidrug resistant
OTI	orotracheal intubation
SAPS-II	simplified acute physiology score II
SARS-CoV-2	severe acute respiratory syndrome-coronavirus 2
VAP	ventilator-associated pneumonia
